# Idiosyncratic responding during movie-watching predicted by age differences in attentional control

**DOI:** 10.1016/j.neurobiolaging.2015.07.028

**Published:** 2015-11

**Authors:** Karen L. Campbell, Meredith A. Shafto, Paul Wright, Kamen A. Tsvetanov, Linda Geerligs, Rhodri Cusack, Cam-CAN, Lorraine K. Tyler, Carol Brayne, Ed Bullmore, Andrew Calder, Rhodri Cusack, Tim Dalgleish, John Duncan, Rik Henson, Fiona Matthews, William Marslen-Wilson, James Rowe, Meredith Shafto, Karen Campbell, Teresa Cheung, Simon Davis, Linda Geerligs, Rogier Kievit, Anna McCarrey, Darren Price, Jason Taylor, Kamen Tsvetanov, Nitin Williams, Lauren Bates, Tina Emery, Sharon Erzinçlioglu, Andrew Gadie, Sofia Gerbase, Stanimira Georgieva, Claire Hanley, Beth Parkin, David Troy, Jodie Allen, Gillian Amery, Liana Amunts, Anne Barcroft, Amanda Castle, Cheryl Dias, Jonathan Dowrick, Melissa Fair, Hayley Fisher, Anna Goulding, Adarsh Grewal, Geoff Hale, Andrew Hilton, Frances Johnson, Patricia Johnston, Thea Kavanagh-Williamson, Magdalena Kwasniewska, Alison McMinn, Kim Norman, Jessica Penrose, Fiona Roby, Diane Rowland, John Sargeant, Maggie Squire, Beth Stevens, Aldabra Stoddart, Cheryl Stone, Tracy Thompson, Ozlem Yazlik, Marie Dixon, Dan Barnes, Jaya Hillman, Joanne Mitchell, Laura Villis, Lorraine K. Tyler

**Affiliations:** a Department of Psychology, University of Cambridge, Cambridge, UK; b MRC Cognition and Brain Sciences Unit, Cambridge, UK; c The Brain and Mind Institute, Western University, London, Ontario, Canada; d Cambridge Centre for Ageing and Neuroscience (Cam-CAN), University of Cambridge and MRC Cognition and Brain Sciences Unit, Cambridge, UK

**Keywords:** Natural vision, Aging, Attentional control, fMRI, Independent components analysis, Intersubject correlation

## Abstract

Much is known about how age affects the brain during tightly controlled, though largely contrived, experiments, but do these effects extrapolate to everyday life? Naturalistic stimuli, such as movies, closely mimic the real world and provide a window onto the brain's ability to respond in a timely and measured fashion to complex, everyday events. Young adults respond to these stimuli in a highly synchronized fashion, but it remains to be seen how age affects neural responsiveness during naturalistic viewing. To this end, we scanned a large (N = 218), population-based sample from the Cambridge Centre for Ageing and Neuroscience (Cam-CAN) during movie-watching. Intersubject synchronization declined with age, such that older adults' response to the movie was more idiosyncratic. This decreased synchrony related to cognitive measures sensitive to attentional control. Our findings suggest that neural responsivity changes with age, which likely has important implications for real-world event comprehension and memory.

## 1 Introduction

Movies have the power to transport your mind from the narrow, impersonal bore of an magnetic resonance imaging (MRI) magnet to a world more synonymous with everyday life, replete with sights, sounds, and language. Despite their complexity, these naturalistic stimuli tend to drive neural activation in the same way across individuals (Hasson et al., 2004, Hasson et al., 2010), suggesting that our experience of real-world events is largely shared. Although responding in the same way as others is not a perquisite for perception, it does seem to reflect the optimal response to a given stimulus, in that asynchronous responding tends to relate to poor comprehension (Hasson et al., 2009) and memory (Hasson et al., 2008a). This may be because synchronized activity reflects shared attention to the most relevant stimulus in the environment, as nominated by the majority. Empirical work supports this view, as (1) participants' eye movements tend to track the same focal item within each shot (Dorr et al., 2010, Hasson et al., 2008b), (2) materials that are rated as more engaging tend to yield the highest degree of neural synchronization (Dmochowski et al., 2014), and (3) disruptions to story narrative, and ergo meaning, tend to reduce overlap across participants (Dmochowski et al., 2012, Hasson et al., 2008b). Although previous work has mainly focused on aspects of the stimulus itself that make it more or less captivating, these findings suggest that individual differences in attentional control should also predict intersubject synchronization. Individuals with greater attentional control should be better able to maintain focus on the movie and should therefore show higher synchronization with others.

Individuals of all ages differ in their ability to control the focus of attention, but on average, this ability tends to decline with age (Hasher and Zacks, 1988). For instance, relative to younger adults, older adults are less able to ignore distracting information (May, 1999), prevent reflexive eye movements toward irrelevant onsets (Campbell and Ryan, 2009), and to sustain attention to a task to produce consistent response times (RTs; Hultsch et al., 2002). They also experience more interference from internally generated distraction, such as competing responses during memory retrieval (Healey et al., 2013), and these intrusive thoughts affect their ability to stay on task, especially as task demands increase (Persson et al., 2007, Sambataro et al., 2010). This suggests that even during task-free, naturalistic viewing, older adults should be less able to sustain attention to a movie and prevent interference from both external (e.g., scanner noise; Stevens et al., 2008) and internal distraction (Mishra et al., 2013). As a result, they should show altered patterns of neural responsiveness and reduced synchronization with others during naturalistic viewing.

To test this hypothesis, we obtained functional magnetic resonance imaging (fMRI) data while participants from a large population-based cohort (aged 18–88 years) watched Alfred Hitchcock's “Bang! You're Dead”, a movie previously shown to yield widespread correlations throughout the cortex (Hasson et al., 2010). Functional networks were derived using independent components analysis (ICA; Beckmann and Smith, 2005, Naci et al., 2014), a data-reduction technique that decomposes the continuous fMRI time series into a set of components (or neural networks), each with an associated spatial map, group-average timecourse, and set of individual timecourses reflecting the level of activation within a given network by a given participant at each time point. A measure of synchronization was then derived for each participant, based on the correlation between their individual timecourse and that of the group.

Given age-related declines in attentional control, we expected older adults' network timecourses to show less synchronization with the group-average timecourse. To test the reproducibility of our main finding of interest (i.e., decreased temporal synchrony with age), we also ran a supplementary region of interest (ROI) analysis looking at intersubject correlations in the raw fMRI timecourses of a large number of small ROIs (Craddock et al., 2012).

Furthermore, we expected intersubject synchronization to be positively related to measures which are sensitive to attentional control. Specifically, we expected higher synchronization to be associated with better performance on a test of fluid intelligence (widely thought to depend on attentional control; Duncan, 2013, Engle et al., 1999, Kane and Engle, 2002), but not on measures of crystallized intelligence (or semantic knowledge). Crystallized intelligence is less dependent on attentional control (Cole et al., 2012) and shows a different life span trajectory (Horn and Cattell, 1967). We also gave participants a speeded reaction time (RT) task, in which they had to respond as quickly as possible to visual cues. Here, we expected higher synchronization to be associated with less variable RTs, rather than faster responding per se, as previous work suggests that RT variability is a stronger predictor of attentional control than mean RT itself (MacDonald et al., 2009, Stuss et al., 2003).

## 2 Methods

### 2.1 Participants

A population-derived sample (N = 221, 18–88 years old, M = 56.23, standard deviation [SD] = 17.73) were recruited as part of the Cambridge Centre for Ageing and Neuroscience project (Shafto et al., 2014). Exclusion criteria included low performance (24 or lower) on the Mini-Mental State Exam (Folstein et al., 1975), poor hearing (failing to hear 35 dB at 1000 Hz in both ears), poor vision (below 20/50 on the Snellen test), poor English knowledge (non-native or nonbilingual English speakers), self-reported substance abuse, and current serious health conditions (e.g., self-reported major psychiatric conditions, current chemotherapy and/or radiotherapy, or a history of stroke). We also excluded people who were not appropriate for MRI or magnetoencephalograph scanning, which included people with safety- and health-contraindications (e.g., disallowed implants, pacemakers, recent surgery or any previous brain surgery, current pregnancy, facial or very recent tattoos, or a history of multiple seizures or fits) as well as comfort-related issues (e.g., claustrophobia or self-reported inability to lay supine for an hour). Demographic information (including age and sex) for this sample is provided in Supplementary Table 1. Informed consent was obtained from all participants and ethical approval for the study was obtained from the Cambridgeshire 2 (now East of England—Cambridge Central) Research Ethics Committee.

### 2.2 Cognitive tasks

Participants performed several cognitive tasks outside the scanner as part of a larger test battery (for a full description, see Shafto et al., 2014), but here, we focus on measures which are sensitive to attentional control (fluid intelligence and RT variability) and control measures which are less dependent on control (crystallized intelligence and mean RT). Our measure of fluid intelligence was the Cattell Culture Fair (Cattell and Cattell, 1960), a timed pen-and-paper test in which participants perform a series of nonverbal puzzles. Crystallized intelligence was measured using the Spot-the-Word Test (Baddeley et al., 1993), in which participants see word-nonword pairs (e.g., pinnace-strummage) and decide which is the real word. Finally, on the speeded choice RT task, participants used a 4-button response box and responded as quickly as possible (maximum 3s) to 1 of 4 possible cued fingers (66 trials, variable inter-trial interval with a mean of 3.7 seconds). Outlier RTs that were >3 standard deviations (SDs) away from an individual's mean were removed (6% of trials on average), and intraindividual means (choice RT_mean_) and SDs (choice RT_ISD_) were calculated using the remaining trials. Data from 34 participants were missing for the choice RT task because of equipment error (final N = 186).

### 2.3 The movie

In the scanner, participants watched an edited version of Alfred Hitchcock's “Bang! You're Dead”, a black and white television drama which has previously been used to study neural synchronization (Hasson et al., 2004). Because of time constraints, the full 25-minute episode was condensed to 8 minutes with the narrative of the episode preserved. Participants were instructed to watch, listen, and pay attention to the movie (they were not aware of its title).

### 2.4 Image acquisition

Imaging was performed on a 3T Siemens TIM Trio System at the MRC Cognition Brain and Sciences Unit, Cambridge, UK. A 3D-structural MRI was acquired for each participant using T1-weighted sequence (Generalized Autocalibrating Partially Parallel Acquisition; repetition time = 2250 ms; echo time = 2.99 ms; inversion time = 900 ms; flip angle α = 9°; matrix size 256 mm × 240 mm × 19 mm; field of view = 256 mm × 240 mm × 192 mm; resolution = 1 mm isotropic; accelerated factor = 2) with acquisition time of 4 minutes and 32 seconds. For the functional scan, T_2_*-weighted echo planar images (EPIs) were acquired using a multiecho sequence (repetition time = 2.47 seconds; 5 echoes [echo time = 9.4 ms, 21.2 ms, 33 ms, 45 ms, 57 ms]; flip angle 78°; 32 axial slices of thickness of 3.7 mm with an interslice gap of 20%; field of view = 192 mm × 192 mm; voxel-size = 3 mm × 3 mm × 4.44 mm) with an acquisition time of 8 minutes and 13 seconds.

### 2.5 Imaging analyses

#### 2.5.1 Preprocessing

Functional and structural images were preprocessed using SPM12 (Wellcome Department of Imaging Neuroscience, University College London, London, UK), as implemented in AA 4.0 pipeline (https://github.com/rhodricusack/automaticanalysis). Fieldmaps were used to undistort the functional EPI images of each participant, which were then motion-corrected and slice-time corrected. The T1 and T2 structural images were coregistered to a Montreal Neurological Institute (MNI) template image, bias-corrected, and then combined to segment various tissue classes (Taylor et al., 2013) using unified segmentation (Ashburner and Friston, 2005). The segmented gray matter images were then used to create a study-specific anatomical template using the DARTEL procedure to optimize inter-participant alignment (Ashburner, 2007), which was then transformed to MNI space. The EPI images were then coregistered to the T1 image and normalized to MNI space using the DARTEL flowfields.

To reduce the effects of head motion, which tends to increase with age and may contribute to age differences in functional connectivity (e.g., Power et al., 2012), we applied a wavelet despiking method that removes motion artifact from fMRI data without deleting frames from the fMRI time series (Patel et al., 2014). This method detects irregular events at different frequencies by identifying chains of outlying wavelet coefficients and removes these from voxel time series. The algorithm can remove both prolonged motion artifacts (such as spin-history effects) as well as higher frequency events (such as spikes). The total amount of despiking performed on a data set is quantified as the average percentage of voxels containing a spike within a frame of data, averaged across the whole run. This spike percentage measure was highly correlated with participants' total motion during the run (r = 0.79), quantified as the root mean square volume-to-volume displacement (Jenkinson et al., 2002). Two participants were excluded from further analyses for having an average spike percentage >3 SDs above the mean (1.75%), leaving a total of 219 participants.

After wavelet denoising, the data were smoothed with an 8-mm full width at half maximum (FWHM) Gaussian kernel. Removal of nonbrain structures was done using the Brain Extraction Tool (Smith, 2002) from the Oxford Centre for Functional Magnetic Resonance Imaging of the Brain's Software Library (FSL version 4.1.8; Smith et al., 2004). All volumes also underwent mean-based intensity normalization (using the 4D grand-mean) and were high-pass filtered (Gaussian-weighted least-squares straight line fitting, equivalent to 100 seconds) to remove low frequency artifacts and resampled to a resolution of 4 mm to reduce the computational burden.

#### 2.5.2 Independent components analysis

We chose to examine intersubject synchronization at the level of network timecourses, rather than individual voxels (Hasson et al., 2004) because age differences in gray matter structure may be particularly problematic for the fine-grained alignment needed for voxelwise correlations across participants (Haxby et al., 2014). All participants' data were simultaneously entered into a tensor-ICA (Beckmann and Smith, 2005), a multivariate analysis technique that identifies patterns of neural activation that are shared across participants over time. This method decomposes the fMRI signal into a set of independent components, each with an associated timecourse (which describes the level of activation in that component over time), spatial map (which describes the brain regions contributing to the activity timecourse), and set of subject modes (i.e., “loading values”, which indicate the degree to which each participant expresses a given spatiotemporal component). An initial analysis indentified 1 participant (aged 45 years) as an outlier (with loading values >3 SD from the mean) and this participant was removed before running the final analysis (N = 218).

**Fig. 1:**
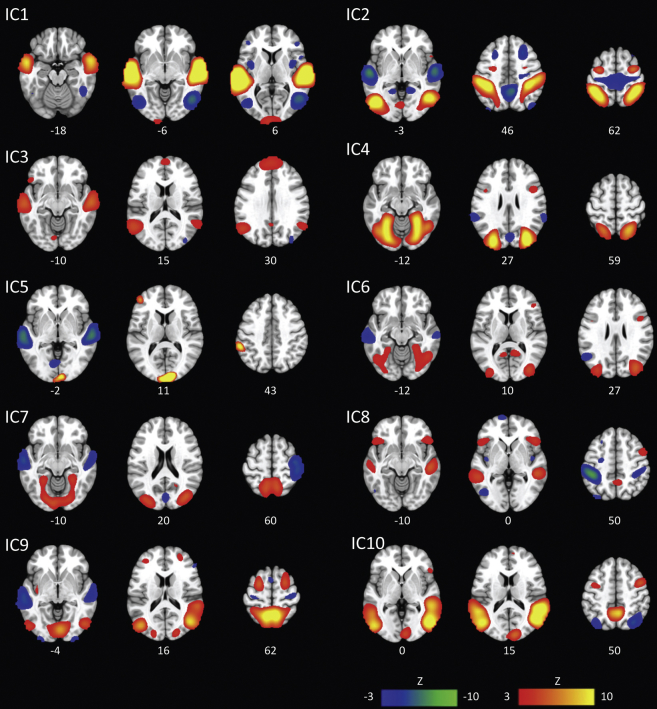
The 10 strongest networks activated during the movie: (IC1) auditory, (IC2) visuospatial, (IC3) language and/or dorsal default mode network (DMN), (IC4) visual, (IC5) posterior salience, (IC6) visual, (IC7) visual and/or ventral DMN, (IC8) language, (IC9) ventral DMN, and (IC10) language and/or ventral DMN networks (labels primarily based on the most highly correlated template from Shirer et al., 2012). Regions loading positively on a component are shown in warm colors, regions loading negatively on a component are shown in cool colors (z-values ranging from ± 3.0–10.0). Montreal Neurological Institute coordinates (in mm) shown below each slice.

**Fig. 2:**
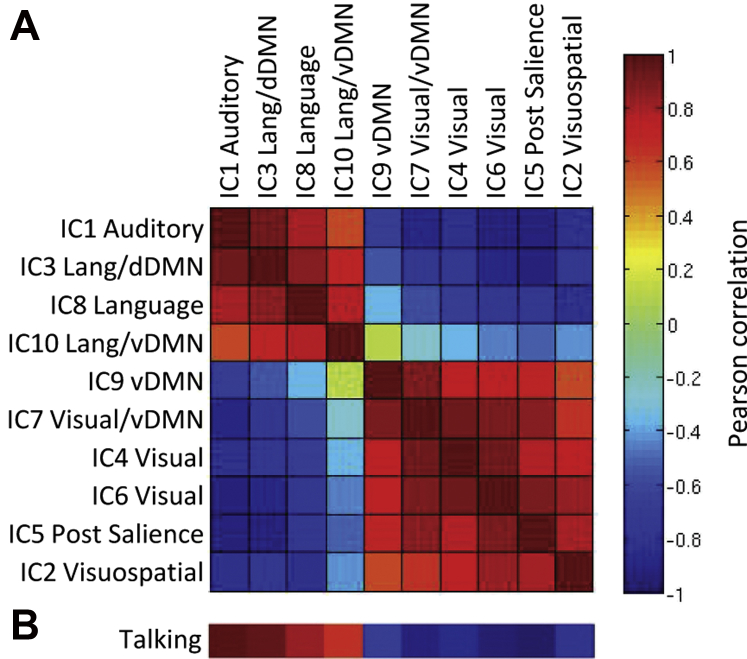
(A) Correlation between pairs of component timecourses clustered into 2 groups: networks primarily related to auditory and language processing and those primarily related to vision and attention. (B) Correlation between component timecourses and talking during the movie. Abbreviations: dDMN, dorsal default mode network; vDMN, ventral default mode network.

The data were decomposed into 56 components using the Laplace approximation of the model order. We selected the 10 most strongly expressed components (i.e., those with the highest median loading values) for further analysis. These components closely resembled established resting state networks (Allen et al., 2011, Shirer et al., 2012) and showed relatively low correlation to white matter and cerebrospinal fluid templates (see Supplementary Table 2). The components are labeled in Fig. 1A according to the most highly correlated template from Shirer et al. (2012); (see Supplementary Table 2 for correlation values). We also calculated the correlation between pairs of component timecourses and these are clustered in Fig. 2A using a k-nearest neighbor algorithm. Finally, to verify that this analysis approach is sensitive to the content of the movie, we first coded all time points during the movie that contained talking and convolved these events with a canonical hemodynamic response function. We then calculated the correlation between this talking timecourse and each of our component timecourses (Fig. 2B).

To examine the effect of age on the expression of the components, we calculated the partial correlation between age and loading value separately for each of the 10 components (controlling for education), with a bootstrap estimate (using 1000 samples) of the 95% confidence interval for each correlation. Highest education obtained (see Supplementary Table 1) was included as a covariate in this and all subsequent analyses to control for potential cohort effects within our sample.

To quantify intersubject synchronization in the temporal dimension, we calculated the correlation between each individual's network timecourse and the group-average timecourse for each of our 10 components separately. To quantify deviation from the mean in the spatial dimension, we first used the dual regression method (Damoiseaux et al., 2012, Filippini et al., 2009) to obtain individual spatial maps for each participant for each component of interest. This method involves (1) using the unthresholded tensor-ICA spatial maps in a linear model fit (spatial regression) against the preprocessed fMRI data, resulting in participant-specific timecourses for each component, and (2) variance normalizing these participant-specific timecourses and using them in a linear model fit (temporal regression) against the preprocessed fMRI data, resulting in participant-specific spatial maps for each component. Then, for each of the 10 components of interest, we correlated each participant-specific map with the original component map to obtain a single measure per participant indicating the degree to which she or he deviates from the group average in the spatial dimension. Correspondence in the temporal and spatial dimensions was then correlated with age, with a statistical threshold of *p* < 0.05 (Bonferroni corrected for the number of tests).

#### 2.5.3 ROI analysis

For the ROI analysis, we parcellated the whole brain into 840 ROIs (average size = 21.8 voxels, SD = 5.8) created by Craddock et al. (2012). We chose this level of parcellation because it should be fine-grained enough to capture regional differences in temporal synchrony but also coarse enough to avoid potential age-bias in the coregistration of functional images (which may be particularly problematic for voxelwise correlations). For each individual, we extracted the mean timecourse for each ROI and correlated this to the mean timecourse of all other participants. These ROI correlation (or synchrony) values were then converted to z-scores, using the Fisher r-z transformation, to obtain a more normal distribution. Subsequently, we correlated the synchrony values in each ROI with age (controlling for education). We also tested whether older adults are more similar to their age-matched peers than they are to the group as a whole. For this analysis, we first selected 2 equally sized groups: older adults (>65 years, n = 81) and younger adults (<50 years, n = 82). We then calculated the correlation between each individual's ROI timecourse and the mean timecourse of their group, and then performed a 2-sample *t* test for each ROI testing the difference in average synchrony between the 2 age groups (again, including education as a covariate). Results shown are thresholded at both *p* < 0.001 and *p* < 0.05/840 (i.e., Bonferroni corrected).

### 2.6 Intersubject synchronization and measures of attentional control

To examine how intersubject synchronization relates to individual differences in attentional control, we first calculated a single synchronization score for each participant as their mean correlation to the group-average timecourse across our 10 components of interest (although a similar pattern of results are seen if we use mean synchronizations across ROIs). For each of our cognitive measures of interest (fluid intelligence, crystallized intelligence, choice RT_mean_, and choice RT_ISD_), we ran a separate regression model, predicting performance from age, synchronization score, and the age × synchronization score interaction (which tests for a change in the relationship between brain and behavior with age). Education was also included as a covariate of no interest. Because RT means and SDs tend to be highly correlated to each other and similarly correlated with age (Hale et al., 1988), we included each of these measures as a covariate in the model predicting the other, which ensured that whatever relationship we observed between synchronization and RT means or SDs would be unique to that measure. Effects were considered significant at a statistical threshold of *p* < 0.05.

## 3 Results

### 3.1 ICA results

The ICA analysis estimated 56 components, each with an associated spatial map, timecourse of activation, and set of participant loading values, which indicate the degree to which each participant expresses a given spatiotemporal pattern. The spatial maps for the 10 most strongly expressed of these components are shown in Fig. 1 and are labeled according to the most highly correlated template(s) from Shirer et al., 2012; (Supplementary Table 2). These components closely resemble previously established resting state networks (Allen et al., 2011, Shirer et al., 2012), correlating most highly with the (1) auditory, (2) visuospatial, (3) language and/or dorsal default mode network (DMN), (4) visual, (5) posterior salience, (6) visual, (7) visual and/or ventral DMN, (8) language, (9) ventral DMN, and (10) language and/or ventral DMN networks. Examining the correlations between pairs of network timecourses, we see that these networks clustered into 2 groups (see Fig. 2A). Auditory and language components were highly correlated during the movie and anticorrelated with visual and attentional networks, whereas the latter networks were highly intercorrelated. Moreover, the auditory and language networks covaried with talking during the movie (Fig. 2B), suggesting that the ICA approach successfully identified neurocognitive networks which correspond to meaningful events within the movie.

**Fig. 3:**
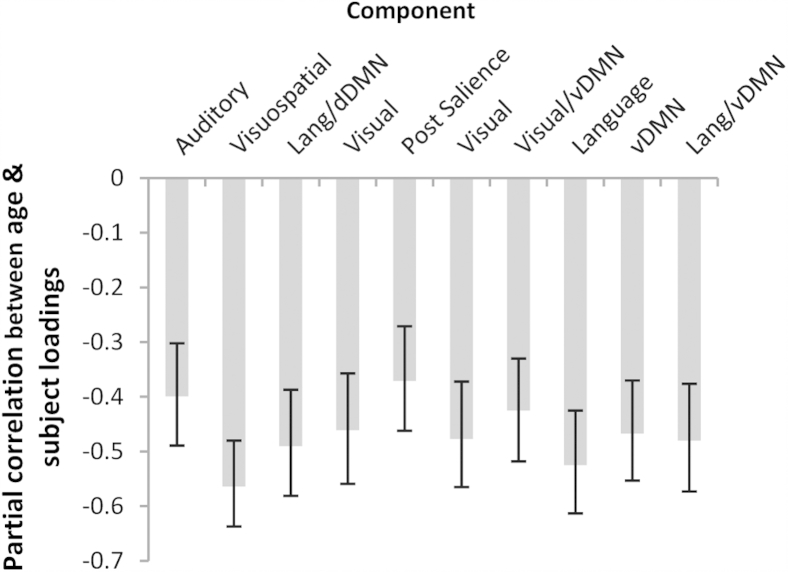
Partial correlation between age and individual loading values (controlling for education) for the 10 components shown in Fig. 1. Error bars represent 95% bootstrap confidence intervals. Abbreviations: dDMN, dorsal default mode network; Lang, language; Post, posterior, vDMN, ventral default mode network.

**Fig. 4:**
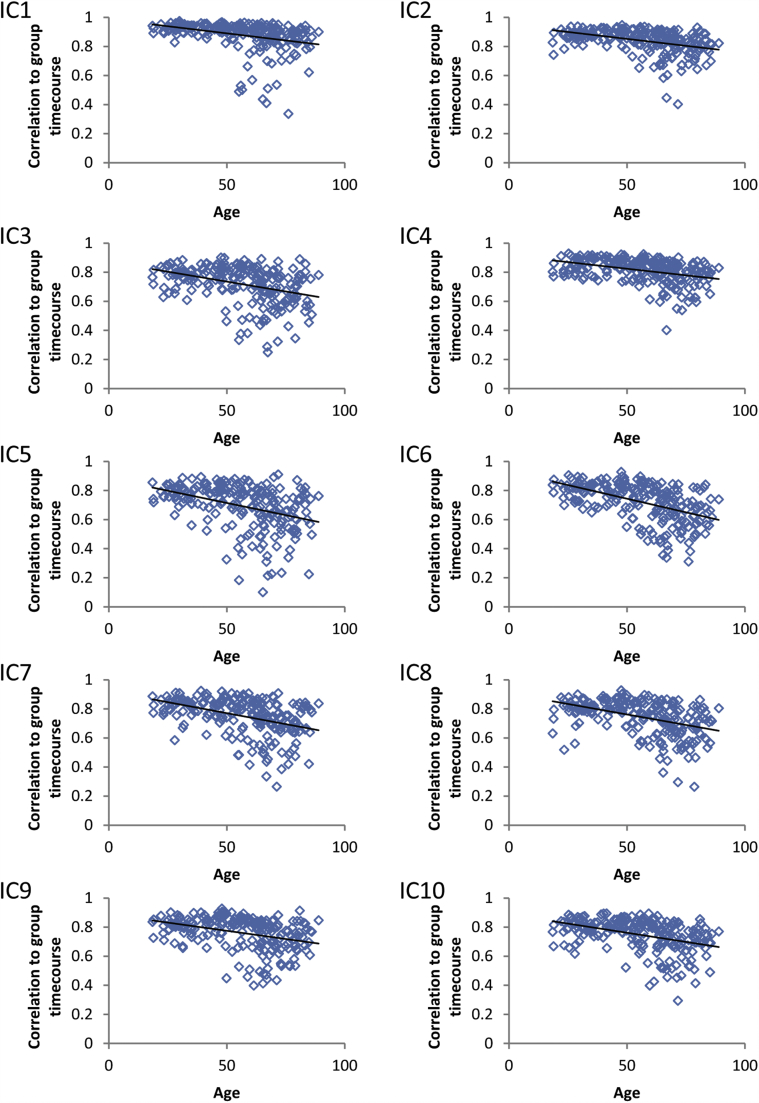
Scatterplots showing the correlation between age and the correlation of individual network timecourses to the group-average timecourse for each of the 10 components of interest shown in Fig. 1. Corresponding correlation values shown in Supplementary Table 3.

**Fig. 5:**
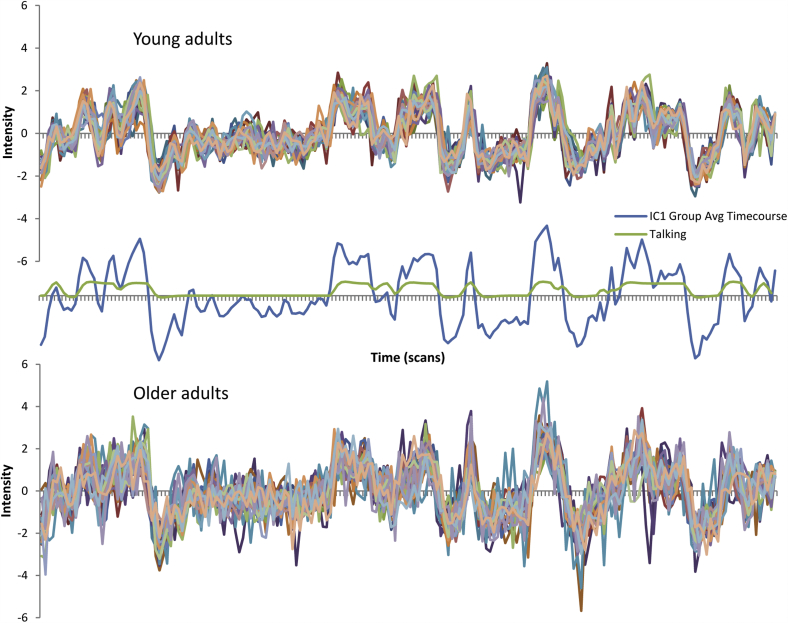
Individual timecourses from the auditory network for the 30 youngest and 30 oldest participants (each line represents a single participant), with the group-average timecourse and the timecourse of talking during the movie plotted in between.

Participant loading values, or network expression, significantly declined with age across all 10 components of interest (Fig. 3). Moreover, and in line with our predictions, intersubject synchronization, or correlation to the group-average timecourse, declined with age across all components, controlling for education (see Fig. 4, Supplementary Table 3). Figure 5 illustrates this for 1 representative component (the auditory network) by plotting the individual timecourses of the 30 youngest and 30 oldest participants, along with the group-average timecourse and the timecourse of talking during the movie. Although younger adults clearly responded in a synchronized way to the movie, older adults were much more variable in their response. This age difference remains even if we partial out the effects of motion (Supplementary Fig. 1), suggesting that it cannot be explained alone by age differences in head motion during the scan (Power et al., 2012, Satterthwaite et al., 2012, Van Dijk et al., 2012).

We also calculated an equivalent metric of deviation from the mean in the spatial dimension, by first using dual regression (Damoiseaux et al., 2012, Filippini et al., 2009) to obtain participant-specific spatial maps and then calculating the correlation between these maps and the original component map, for each component of interest separately. As with the temporal dimension, correlation to the group-average spatial map declined with age across all components (see Supplementary Table 3), although the absolute decline in spatial correlation across the life span was quite small (averaged across the 10 networks, correlations declined from 0.78 in decile 1 to 0.74 in decile 7; see Supplementary Fig. 2).

### 3.2 ROI analysis results

**Fig. 6:**
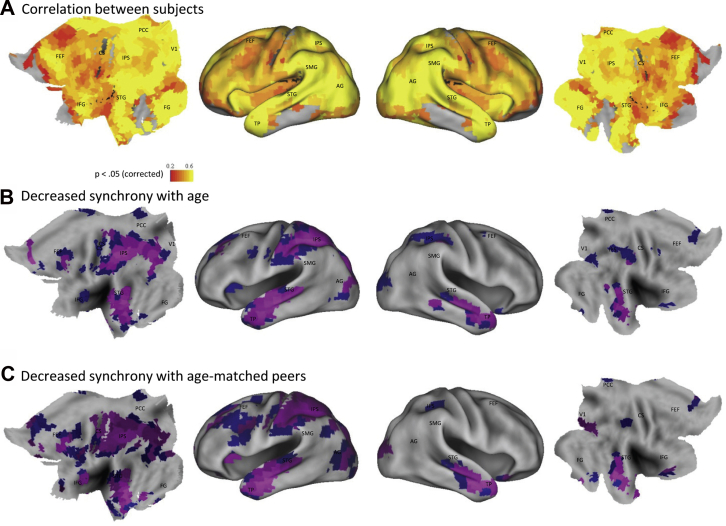
Intersubject synchrony results from the region of interest analysis. Significant intersubject correlations were seen throughout the cortex [(A); Bonferroni corrected for multiple comparisons]. Synchronization was negatively correlated with age (controlling for education) in several regions [(B); blue thresholded at *p* < 0.001; violet regions survive Bonferroni correction, *p* < 0.05/840]. A similar pattern of results is seen panel C if younger (<50 years) and older adults (>65 years) are instead correlated to their age-matched peers and a group contrast is performed with education as a covariate (young > old, blue *p* < 0.001, violet *p* < 0.05/840), suggesting that older adults are just as dissimilar from each other as they are to younger adults. (For interpretation of the references to color in this figure legend, the reader is referred to the Web version of this article.)

To ensure that the observed age-related decrease in intersubject synchronization was not due to a difference in the network structure of older adults, or specific to the method we applied, we ran a supplementary ROI analysis. To this end, we extracted the mean timecourse from 840 ROIs (Craddock et al., 2012) and then for each ROI separately, calculated the correlation between an individual's timecourse and the mean timecourse of all other participants. Similar to previous work using a voxelwise approach (Hasson et al., 2004), this method yielded robust intersubject correlations throughout the cortex (Fig. 6A), with the strongest synchronization in primary visual and auditory regions and weaker synchronization in sensorimotor and rostral frontal cortex. Importantly, we replicate the main finding of interest from our ICA analysis, showing that intersubject correlations declined with age (after controlling for education) in several regions (Fig. 6B), including middle occipital cortex, intraparietal sulcus, the temporal poles, anterior cingulate, and left superior frontal lobe. There were no regions in which synchrony increased with age. Furthermore, if we split the sample into 2 groups (younger and older), correlate individuals to their age-matched peers, and then compare the strength of these correlations between groups (including education as a covariate), a very similar pattern of results emerge (Fig. 6C), suggesting that older adults are just as dissimilar to each other as they are to younger adults. Taken together, these findings suggest that intersubject synchronization during naturalistic viewing declines with age.

### 3.3 Intersubject synchronization and measures of attentional control

Means and SDs for the cognitive measures are shown per decile in Supplementary Table 1. As expected from previous findings, aging was associated with lower performance on tests sensitive to attentional control, with older adults scoring lower on the Cattell test of fluid intelligence (r = −0.65, *p* < 0.001) and more variably (r = 0.58, *p* < 0.001), as well as slower (r = 0.65, *p* < 0.001), on the Choice RT task, all controlling for education. In contrast, crystallized intelligence increased with age, controlling for education (r = 0.22, *p* < 0.01).

**Fig. 7:**
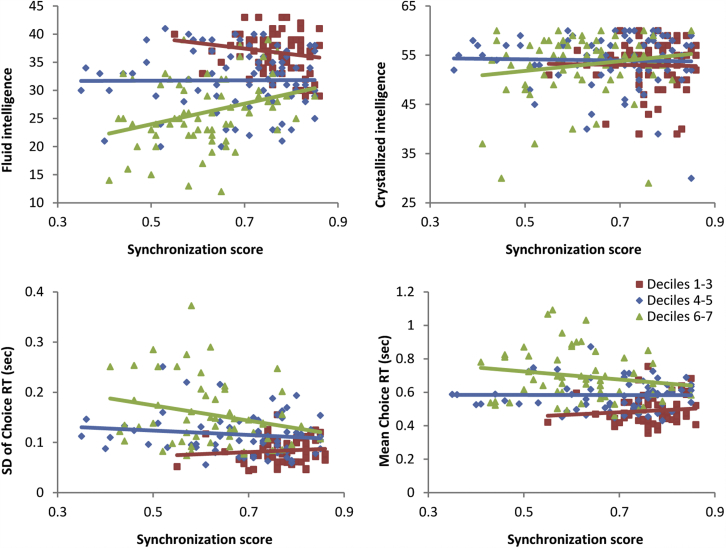
Scatterplots showing the relationship between intersubject synchronization and measures sensitive to attentional control (i.e., fluid intelligence and RT variability), as well as control measures less dependent on attentional control (i.e., crystallized intelligence and mean RT). To show how this relationship changed with age in some cases, data are split into 3 roughly equal subgroups: deciles 1–3 (red, N = 72), deciles 4–5 (blue, N = 81), and deciles 6–7 (green, N = 65). Abbreviations: RT, reaction time; SD, standard deviation. (For interpretation of the references to color in this figure legend, the reader is referred to the Web version of this article.)

To test whether individuals with greater attentional control show higher synchronization with the group during the movie, we first calculated a single synchronization score for each participant as their mean correlation to the group-average timecourse across the 10 components from our ICA analysis. We then performed separate regression analyses for each of the cognitive measures of interest (i.e., fluid intelligence and choice RT_ISD_ as proxies for attentional control; crystallized intelligence, and choice RT_mean_ as control measures, not expected to relate to synchronization). Our predictor variables were age, synchronization score, and the age × synchronization interaction, with education as a covariate. Results from these regressions are shown in Table 1 and scatter plots depicting the relationship between synchronization and each cognitive variable (subdivided into decile subgroups for visualization purposes) are shown in Fig. 7. Higher intersubject synchronization related to lower choice RT_ISD_ across the entire sample (indicated by the significant main effect of synchronization in Table 1) and to higher fluid intelligence among middle-to older-aged adults (indicated by the significant interaction between age and synchronization in Table 1). In contrast, synchronization was not related to crystallized intelligence and, if anything, related positively to choice RT_mean_ due to slightly slower responding by young adults who showed greater synchrony with the group (Fig. 7). These findings suggest that individual differences in attentional control relate to intersubject synchronization during natural viewing, particularly among older adults.

## 4 Discussion

Using both an ICA and ROI-based approach, we showed that intersubject synchronization declines with age during naturalistic viewing, such that older adults respond to a common, driving stimulus in a more variable fashion than younger adults. From the ROI analysis, we see that this decreased synchrony was particularly pronounced in regions responsible for attentional control (i.e., superior frontal lobe and intraparietal sulcus) and language processing (i.e., bilateral middle temporal gyrus and left inferior frontal gyrus). We went on to show that intersubject synchronization was also selectively related to measures which are sensitive to attentional control, such as fluid intelligence and RT variability (Duncan, 2013, Engle et al., 1999, Hasher et al., 2007, Hultsch et al., 2002) but not measures which are less reliant on top-down control, such as crystallized intelligence (Cole et al., 2012). These findings suggest that neural responsivity, or the ability to respond in a timely and appropriate fashion to ongoing events in the environment, differs with age, possibly due to altered patterns of attention during the perception of complex, naturalistic scenes.

To respond to a driving stimulus in the same way as everyone else, as a starting point, one needs to attend to the same things as everyone else. Hollywood, or in this case Hitchcock, makes this easy for us by constructing stimuli which draw attention (as measured by eye movements) to the focal item in each shot (Dorr et al., 2010, Hasson et al., 2008b). When the stimulus is less engaging, such as footage of everyday activities (Hasson et al., 2010) or less popular Super Bowl commercials (Dmochowski et al., 2014), people show less overlap in their neural activation, suggesting that a stimulus needs to be sufficiently captivating to drive attention in this bottom-up manner. However, bottom-up attention is not sufficient; top-down control is also needed, at the very least, to maintain fixation on the movie and limit attention to that information which is most relevant to the plot.

In line with this view, we found that individual differences in attentional control predict intersubject synchronization and on average, temporal synchrony declines with age. We argue that this age-related decline likely reflects a difference in top-down control (Hasher and Zacks, 1988), rather than bottom-up capture, as attentional capture is known to be relatively preserved with age (Folk and Hoyer, 1992, Hartley et al., 1990, Pratt and Bellomo, 1999). Moreover, materials with emotional content tend to be particularly salient to, and better remembered by, older adults (Carstensen and Turk-Charles, 1994, Rahhal et al., 2002) and thus, their attention should have been sufficiently captured by the movie, which contained a lot of emotional content being directed by “The Master of Suspense” himself. In future, measures of attentional focus (Naci et al., 2014) and eye tracking (e.g., Dorr et al., 2010) should be collected at the same time as scanning to better characterize age differences in attentional focus to the movie itself, as this is a caveat of the current design.

Although responding to a movie in an idiosyncratic or variable fashion may, in itself, seem innocuous, our behavioral results suggest that this type of erratic responding is, instead, indicative of attentional dysfunction. What are the consequences of this erratic responding for narrative comprehension and memory? Related behavioral work suggests that older adults' idiosyncratic responding may have consequences for the encoding of temporal order information (Sargent et al., 2013). In these studies, participants typically watch a movie and indicate whenever they think an event boundary has occurred. Older adults tend to vary more in where they draw those boundaries, with those who respond in the most variable or idiosyncratic way being least able to recall the order of events (Bailey et al., 2013, Zacks et al., 2006). Thus, there is something to be said for normative responding, as shared attention appears to track whatever information is most informative in a given scene. Those who stray from the pack ultimately arrive at a different perspective—one that is lacking in key details but may be potentially rich in other, unforeseen ways (Kemper et al., 1990).

Beyond attentional control, these results also contribute to the growing literature on age differences in functional connectivity by showing that aging affects network responsiveness during natural viewing. Watching the movie activated a set of networks similar to canonical resting state networks, in line with previous work showing little change in the spatial extent of large-scale networks moving from rest to task (Betti et al., 2013, Greicius et al., 2003, Smith et al., 2009) and replicated previous demonstrations of an age-related decline in network expression. In fact, the age-related decline observed here was particularly pronounced, in that it spanned across all networks rather than being limited to a few, as has been reported previously in ICA analyses of resting state data (Damoiseaux et al., 2008, Mowinckel et al., 2012). This may be because our use of a common driving stimulus allowed for the quantification of deviation from the mean in both the temporal and spatial dimension (only the latter is possible during rest). However, it should be noted that the observed age differences may be due, at least in part, to cohort differences within our cross-sectional sample. We included education as a covariate in all analyses to help ameliorate these effects, but determining the true effect of age on network responsivity requires further longitudinal testing (e.g., Raz and Lindenberger, 2011), which we hope to do in future.

In conclusion, the present results show that age affects network responsivity and synchronization during naturalistic viewing which, owing to its complex and multimodal nature, is arguably more reflective of everyday life than standard experimental tasks. These findings suggest that as we age, our experience of the world becomes increasingly individualistic, differing not only from those who differ from us in age, but also from our age-matched peers.

## Disclosure statement

The authors have no conflicts of interest to disclose.

## Figures and Tables

**Table 1:** Regressions predicting attentional control as a function of age, education, synchronization score, and the age by synchronization score interaction

Outcome variable	Predictor variables	Model *R*^*2*^	β [95% CI]	SE β	t	*p*
Fluid intelligence		0.56				
	Age^a^		−0.23 [−0.27, −0.19]	0.02	−10.63	<0.0001
	Synchronization		1.86 [−6.20, 9.91]	4.09	0.45	0.65
	Age × synchronization^a^		0.55 [0.03, 1.08]	0.27	2.09	<0.05
	Education^a^		1.79 [1.14, 2.44]	0.33	5.43	<0.0001
Crystallized intelligence		0.19				
	Age^a^		0.08 [0.04, 0.13]	0.02	3.64	<0.001
	Synchronization		5.59 [−2.44, 13.63]	4.08	1.37	0.17
	Age × synchronization		0.002 [−0.51, 0.52]	0.26	0.006	0.99
	Education^a^		2.30 [1.52, 3.09]	0.40	5.76	<0.0001
Choice RT_ISD_		0.66				
	Age		0.0002 [−0.0002, 0.0006]	0.0002	0.85	0.40
	Synchronization^a^		−0.07 [−0.12, −0.02]	0.03	−2.64	<0.01
	Age × synchronization		−0.001 [−0.005, 0.002]	0.002	−0.75	0.46
	Choice RT_mean_^a^		0.28 [0.19, 0.37]	0.05	6.05	<0.0001
	Education		0.0002 [−0.005, 0.005]	0.003	0.08	0.93
Choice RT_mean_		0.70				
	Age^a^		0.003 [0.002, 0.004]	0.0005	4.84	<0.0001
	Synchronization^a^		0.16 [0.04, 0.28]	0.06	2.70	<0.01
	Age × synchronization		−0.005 [−0.02, 0.006]	0.006	−0.88	0.38
	Choice RT_ISD_^a^		1.60 [1.25, 1.92]	0.17	9.34	<0.0001
	Education		−0.004 [−0.02, 0.009]	0.007	−0.67	0.51

Statistical models were computed separately for each cognitive measure. Results represent regression parameters for a given cognitive task predicted by age, education, synchronization score, and the age × synchronization score interaction. To control for the high degree of correlation between RT_mean_ and RT_ISD_, the model predicting choice RT_ISD_ included choice RT_mean_ as a covariate and the model predicting choice RT_mean_ included choice RT_ISD_ as a covariate. Beta values reflect unstandardized regression coefficients.

Key: CI, confidence interval; ISD, intraindividual standard deviation; RT, reaction time; SE, standard error.

a Significant predictors.
