# Supplementary material for: Idiosyncratic responding during movie-watching predicted by age differences in attentional control
Source: Neurobiol Aging. 2015 Nov;36(11):3045–55. doi: 10.1016/j.neurobiolaging.2015.07.028 (PMC4706158; doi:10.1016/j.neurobiolaging.2015.07.028)

*Supplementary Table 1*. Participant demographics and mean cognitive performance

| **Decile** | | 1 | 2 | 3 | 4 | 5 | 6 | 7 |
| --- | --- | --- | --- | --- | --- | --- | --- | --- |
| ***N*** | | 15 | 30 | 27 | 38 | 43 | 39 | 26 |
| **Age range (years)** | | 18 – 27 | 28 – 37 | 38 – 47 | 48 – 57 | 58 – 67 | 68 – 77 | 78 – 87 |
| **Sex (male/female)** | | 6/9 | 17/13 | 13/14 | 18/20 | 18/25 | 21/18 | 15/11 |
| **Highest Education** |  |  |  |  |  |  |  |  |
|  | University | 11 | 25 | 17 | 30 | 26 | 16 | 14 |
|  | A' Levels | 1 | 3 | 3 | 3 | 5 | 2 | 0 |
|  | GCSE grade | 3 | 1 | 7 | 2 | 10 | 12 | 6 |
|  | None over 16 | 0 | 1 | 0 | 3 | 2 | 9 | 6 |
| **MMSE** |  | 29.09(1.4) | 29.30(1.2) | 29.19(1.3) | 29.08(1.2) | 28.89(1.2) | 28.51(1.3) | 27.85(1.6) |
| **Crystallized intelligence** |  | 51.93(4.9) | 51.93(5.6) | 54.00(3.9) | 54.81(5.9) | 53.81(4.9) | 51.36(7.6) | 55.38(3.4) |
| **Fluid intelligence** |  | 39.07(2.4) | 37.27(3.3) | 34.78(4.1) | 34.82(4.4) | 29.63(4.9) | 26.90(6.7) | 23.96(5.1) |
| **Choice RT_mean_ (sec)** |  | .44 (.05) | .46 (.07) | .54 (.08) | .55 (.07) | .61 (.10) | .65 (.12) | .76 (.17) |
| **Choice RT_ISD_ (sec)** |  | .07 (.01) | .08 (.02) | .10 (.02) | .10 (.03) | .13 (.05) | .14 (.05) | .17 (.08) |

Note. Demographic information are for the final sample of N = 218 (after removal of 3 outliers, see Methods for details). Values in parentheses are standard deviations. MMSE = mini mental status examination; Crystallized intelligence = Spot-the-Word test; Fluid intelligence = Cattell culture fair test; Choice RT_ISD_ = intraindividual SD on the choice RT task.

*Supplementary Table 2*. Correlation between component spatial maps and resting state and tissue class templates

| **Component** | **IC1** | **IC2** | **IC3** | **IC4** | **IC5** | **IC6** | **IC7** | **IC8** | **IC9** | **IC10** |
| --- | --- | --- | --- | --- | --- | --- | --- | --- | --- | --- |
|  |  |  |  |  |  |  |  |  |  |  |
| **Shirer et al. (2012) templates** |  |  |  |  |  |  |  |  |  |  |
| Auditory | **0.22** | 0.03 | 0.00 | -0.02 | 0.03 | -0.02 | -0.02 | 0.07 | -0.02 | 0.01 |
| Basal Ganglia | -0.01 | -0.05 | -0.01 | -0.02 | -0.04 | -0.04 | -0.05 | -0.04 | -0.04 | -0.04 |
| Language | 0.12 | -0.01 | **0.23** | 0.04 | 0.08 | 0.10 | 0.05 | **0.18** | 0.09 | **0.30** |
| Left Executive Control | -0.03 | 0.02 | 0.05 | 0.02 | -0.02 | 0.05 | -0.04 | -0.06 | 0.01 | 0.03 |
| Precuneus | -0.03 | 0.02 | -0.02 | 0.02 | -0.03 | 0.00 | -0.02 | -0.02 | 0.03 | 0.01 |
| Right Executive Control | -0.05 | 0.03 | 0.08 | -0.03 | 0.05 | 0.02 | -0.01 | 0.04 | -0.02 | -0.04 |
| Sensorimotor | -0.05 | -0.01 | -0.06 | -0.03 | -0.06 | -0.01 | 0.05 | -0.02 | -0.01 | -0.01 |
| Anterior Salience | -0.01 | -0.04 | 0.02 | -0.03 | -0.01 | 0.02 | 0.00 | 0.00 | 0.02 | -0.01 |
| Dorsal Default Mode | -0.03 | 0.02 | 0.11 | -0.03 | -0.03 | -0.03 | -0.07 | 0.00 | -0.08 | -0.04 |
| Higher Visual | 0.01 | 0.10 | -0.02 | **0.33** | 0.02 | **0.16** | **0.18** | 0.04 | 0.04 | 0.03 |
| Posterior Salience | 0.00 | 0.17 | 0.01 | 0.01 | **0.12** | 0.00 | 0.03 | 0.11 | 0.11 | 0.07 |
| Primary Visual | -0.02 | -0.01 | -0.04 | 0.01 | 0.05 | 0.01 | 0.06 | 0.03 | -0.02 | -0.02 |
| Ventral Default Mode | -0.03 | 0.10 | 0.07 | 0.08 | -0.06 | 0.09 | **0.18** | 0.04 | **0.28** | 0.11 |
| Visuospatial | -0.02 | **0.30** | -0.06 | 0.10 | -0.04 | 0.02 | 0.05 | 0.14 | 0.04 | 0.01 |
|  |  |  |  |  |  |  |  |  |  |  |
| **Tissue Class Templates** | | | | |  |  |  |  |  |  |
| csf | -0.07 | -0.07 | 0.02 | -0.06 | -0.04 | -0.06 | -0.03 | -0.03 | 0.00 | -0.06 |
| grey | 0.14 | 0.15 | 0.23 | 0.16 | 0.17 | 0.21 | 0.17 | 0.18 | 0.14 | 0.17 |
| white | -0.05 | -0.05 | -0.18 | -0.07 | -0.09 | -0.10 | -0.09 | -0.09 | -0.10 | -0.07 |

Note. Values reflect Pearson correlations between each template and the unthresholded component spatial maps (using absolute values to take positive and negative loading regions into account).

*Supplementary Table 3*. Partial correlation between age and correspondence to the group-average timecourse and spatial map for each component (controlling for education).

| Component | Partial correlation between Age & Correlation to the  Group-Average Timecourse | |  | Partial correlation between Age & Correlation to the Group-Average Spatial Map | |
| --- | --- | --- | --- | --- | --- |
|  | *r* [95% CI] | *p* |  | *r* [95% CI] | *p* |
|  |  |  |  |  |  |
| Auditory | -0.36 [-0.43, -0.29] | < .0001 |  | -0.23 [-0.32, -0.12] | < .0001 |
|  |  |  |  |  |  |
| Visuospatial | -0.42 [-0.50, -0.34] | < .0001 |  | -0.47 [-0.56, -0.37] | < .0001 |
|  |  |  |  |  |  |
| Language/dorsal DMN | -0.37 [-0.46, -0.28] | < .0001 |  | -0.36 [-0.47, -0.25] | < .0001 |
|  |  |  |  |  |  |
| Visual | -0.40 [-0.49, -0.31] | < .0001 |  | -0.37 [-0.46, -0.26] | < .0001 |
|  |  |  |  |  |  |
| Posterior salience | -0.39 [-0.48, -0.30] | < .0001 |  | -0.28 [-0.40, -0.16] | < .0001 |
|  |  |  |  |  |  |
| Visual | -0.52 [-0.59, -0.44] | < .0001 |  | -0.36 [-0.47, -0.24] | < .0001 |
|  |  |  |  |  |  |
| Visual/ventral DMN | -0.43 [-0.52, -0.34] | < .0001 |  | -0.45 [-0.54, -0.35] | < .0001 |
|  |  |  |  |  |  |
| Language | -0.42 [-0.51, -0.32] | < .0001 |  | -0.59 [-0.66, -0.51] | < .0001 |
|  |  |  |  |  |  |
| Ventral DMN | -0.35 [-0.45, -0.26] | < .0001 |  | -0.42 [-0.51, -0.33] | < .0001 |
|  |  |  |  |  |  |
| Language/ventral DMN | -0.41 [-0.49, -0.33] | < .0001 |  | -0.31 [-0.42, -0.19] | < .0001 |

Note. Confidence intervals (95% CI) were derived using a bootstrap estimation with 1000 samples. Corresponding scatterplots shown in Figure 3 and Supplementary Figure 2. DMN = default mode network. All correlations survive Bonferroni correction.

*Supplementary Figure 1*. Partial correlation between age and (A) individual loading values and (B) correspondence to the group-average timecourse for each of the ten components of interest controlling for education and head motion (i.e., RMS volume-to-volume displacement). Error bars represent 95% bootstrap confidence intervals; Lang = language, dDMN = dorsal default, Post = posterior, vDMN = ventral default.


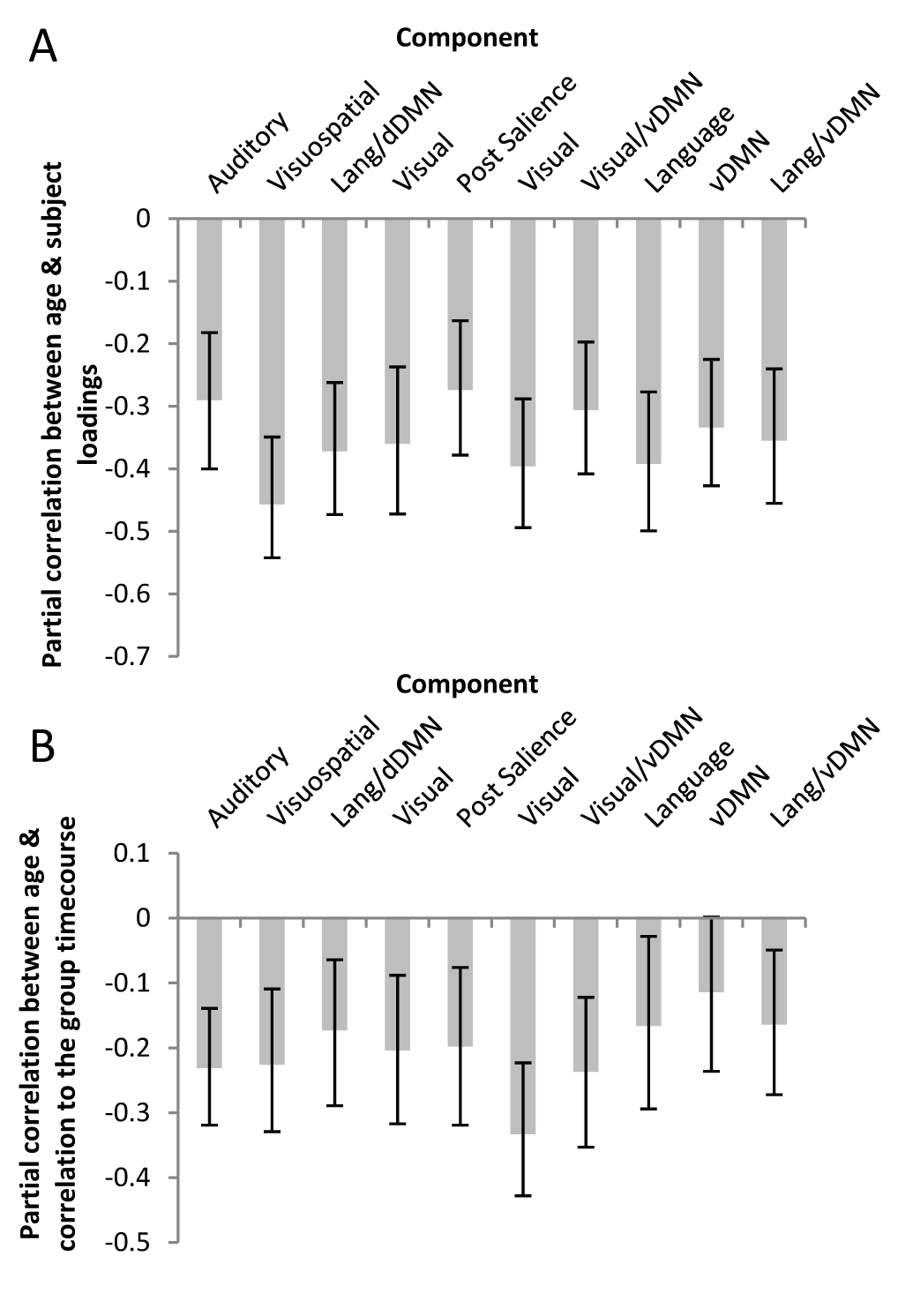


*Supplementary Figure 2*. Scatterplots showing the correlation between age and the correlation of individual spatial maps to the group-average spatial map for each of the ten components of interest shown in Figure 1. Corresponding correlation values shown in Supplementary Table 3.


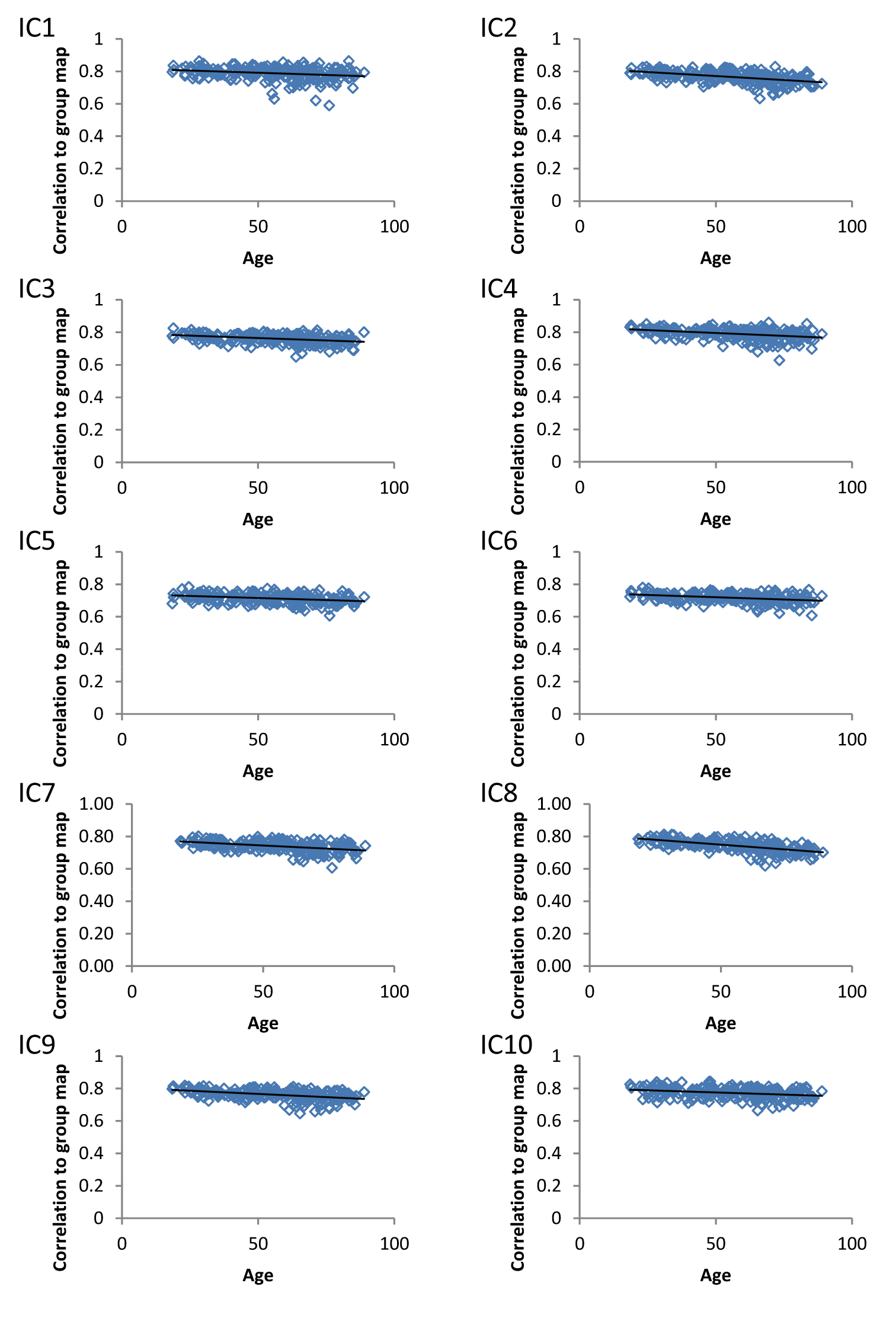

Supplement: Supplementary Tables 1–3 and Supplementary Figs. 1 and 2 [file mmc1.docx]
